# Trained and ready - the case for an inflammatory memory for hematopoietic stem and progenitor cells in the AML niche

**DOI:** 10.18632/oncotarget.28642

**Published:** 2024-09-04

**Authors:** Ding-Wen Chen, Eric K. Wafula, Peter Kurre

**Affiliations:** ^1^Department of Pediatrics, Comprehensive Bone Marrow Failure Center, Division of Hematology, Children’s Hospital of Philadelphia, PA 19104, USA; ^2^Department of Biomedical and Health Informatics, Children's Hospital of Philadelphia, PA 19104, USA; ^3^Perelman School of Medicine, University of Pennsylvania, Philadelphia, PA 19104, USA

**Keywords:** hematopoietic stem and progenitor cells, acute myeloid leukemia, inflammation, innate immune reprogramming, trained immunity

## Abstract

Lifelong hematopoiesis is sustained by crosstalk between hematopoietic stem and progenitor cells (HSPCs) and specialized bone marrow niches. Acute myeloid leukemia (AML) upends that balance, as leukemic blasts secrete factors that remodel the bone marrow into a self-reinforcing leukemic niche. The inflammatory secretome behind this compartmental adaptation accounts for a progressive decline in hematopoietic function that leads to diagnosis and persists through early treatment. Not surprisingly, the mediators of an acute inflammatory injury and HSPC suppression have attracted much attention in an effort to alleviate morbidity and improve outcomes. HSPCs typically recover during disease remission and re-expand in the bone marrow (BM), but little is known about potentially lasting consequences for stem cells and progenitors. We recently showed that AML-experienced HSPCs actively participate in the inflammatory process during leukemic progression. HSPCs are constituent components of the innate immune system, and elegant studies of infection and experimental inflammation over the past decade have described the generation of an adoptively transferable, innate immune memory. Building on this paradigm, we discuss the potential translational relevance of a durable legacy in AML-experienced HSPC.

## INTRODUCTION

**Figure 1:**
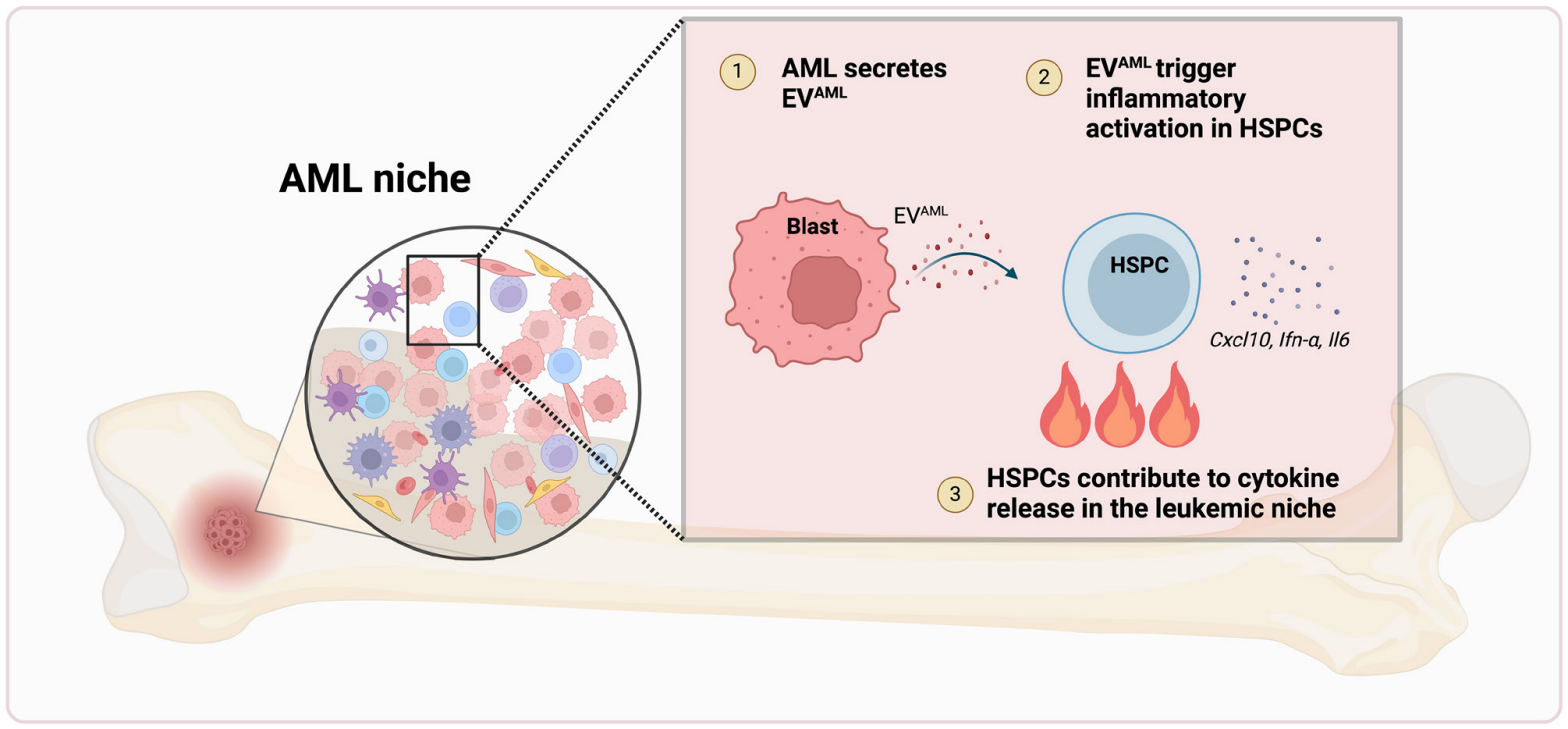
HSPCs exhibit an inflammatory active state in the AML niche. Illustration created using BioRender.

Acute myeloid leukemia (AML) is a heterogenous disease emerging from mutations in hematopoietic stem and progenitor cells (HSPCs) in the bone marrow (BM). As disease progresses, crosstalk between the malignant clones and other cells in the BM microenvironment shapes disease pathogenesis. Inflammation, an enabling characteristic and emerging hallmark of cancer [1], has been implicated in various hematologic malignancies, including AML, and a recent study suggests a positive correlation with severity and patient prognosis [2]. Evidently, a better understanding of the role inflammation plays in AML and how it impacts the BM niche is imperative. As disease progresses, AML blasts [3] and stromal cells [4, 5] are typically considered the two predominant pro-inflammatory cytokine-producing cell populations in the leukemic niche. Healthy HSPCs have been conventionally viewed as bystanders and targets of functional suppression [6]. In this research perspective, we discuss recent work from our lab describing an active role of HSPCs in AML and the potential implications [7] (Figure 1).

### Inflammatory crosstalk between AML blasts and healthy HSPCs in the BM

Xenograft leukemia models have proved useful in modeling leukemic pathophysiology *in vivo*. However, the extent to which studies in immunodeficient recipients reflect signaling in the human AML niche is likely limited [3]. Given this consideration, we recently utilized two immune-competent murine models (C1498 [8] and iMLL-AF9 [9]), to investigate AML crosstalk in a fully functional immune landscape absent in xenograft models. Through initial BM plasma cytokine profiling, we observed elevated pro-inflammatory cytokines, including *Cxcl10*, in the leukemic BM microenvironment, which we validated in human BM plasma from AML patient samples. Then, utilizing single-cell RNA-Seq, we found that the major BM HSPC subpopulations, including long-term hematopoietic stem cells (HSCs), express inflammatory gene set signatures even at low BM leukemic burden, suggesting the involvement of a potent paracrine signaling mechanism [10]. Indeed, we found that AML-derived extracellular vesicles (EV^AML^) accounted for inflammatory signals in healthy HSPCs, including increased interferon-gamma related signals: interferon-stimulated gene 15 (*Isg15*) and C-X-C motif chemokine ligand 10 (*Cxcl10)*. The translational relevance of our finding was validated *ex vivo* by subjecting BM human CD34 cells to human EV^AML^ (hEV^AML^) from MOLM-14, U937, and HL-60 cell lines, which uniformly elicited inflammatory activity. The underlying regulatory cascade is likely complex, but appears to involve both mammalian target of rapamycin (mTOR) and MYC pathways. While the mechanism remains under investigation, the study shows for the first time that HSPCs actively participate in the inflammatory conversion of the AML niche.

### Consequences of inflammatory recruitment of HSCs in AML

Inflammation has proved to be a crucial regulator of HSCs throughout life. Beginning with embryonic development, inflammatory signaling plays an important role in HSC emergence from hemogenic endothelium [11], specification of definitive HSCs and perinatal phenotype transition [12]. Throughout adult life, inflammation regulates stem cell self-renewal and differentiation to support multi-lineage blood cell production during homeostasis [13] and in responding to injury [14]. However, while inflammation is vital for stem and progenitor cells to maintain hematopoietic health, persistent inflammatory conditions are detrimental. For example, rheumatoid arthritis [15], lupus [16], periodontitis [17] and atherosclerosis[18] all trigger stem cell cycling, promote myeloid-bias and boost cytokine output that fuels disease progression. In other words, inflammation is a self-limiting protective tissue response, but when it persists, HSC function capacity is compromised [19, 20].

Like the above debilitating chronic disorders, hematologic malignancies are also inherently inflammatory. Systemically and locally, cancer associated inflammation amplifies the fitness advantage of some malignant clones over others, contributes to chemotherapy resistance [21], and fuels the pathogenesis of myeloproliferative neoplasms [22] and AML [23]. Sustained inflammation can also lead to genetic instability and promote selection and expansion of clones defined by recurring genetic lesions (e.g. DNMT3A, TET2, ASXL1 and others). Clonal hematopoiesis (CH) denotes an increased risk of systemic inflammatory sequelae and progression to hematological malignancies [24].

Several recent studies report that inflammation-experienced HSPCs can be imprinted with a durable memory of inflammation through a mechanism termed trained immunity. Models of experimental infection, vaccination or sterile inflammation indicate that these inflammation-experienced HSPCs exhibit amplified responses to subsequent inflammatory activation [25]. Such an innate immune memory may aggravate disease manifestations of periodontitis [26] and atherosclerosis [27], and has been linked with CH in HSPCs [28].

Considering the abundant evidence for AML associated inflammation, and the involvement of healthy HSPCs discussed in our study, the question arises whether sterile cancer-associated inflammation also has long-term functional consequences. More specifically, does sterile inflammation in the AML BM reprogram residual healthy HSPCs for aggravated recall responses?

**Figure 2:**
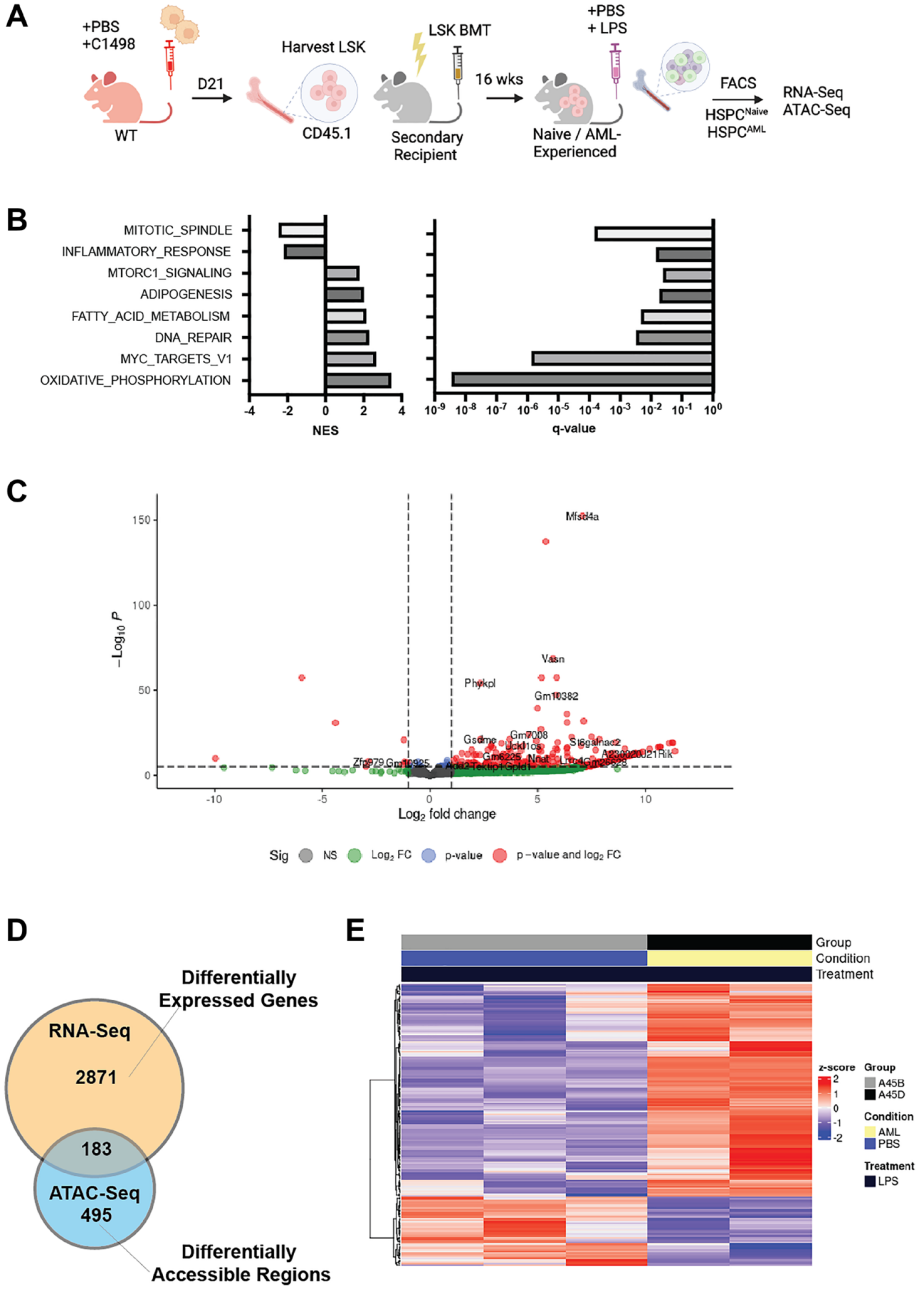
AML-experienced HSPCs (HSPC^AML^) acquire a deregulated transcriptome. (**A**) Schematic of the experimental workflow involving adoptive transfer of FACS-purified HSPC^AML^ (Lin^-^ cKit^+^ Sca1^+^) into conditioned C57BL/6J recipients. LSKs from PBS-injected mice serve as control (HSPC^Naive^). After 16 weeks, HSPCs were analyzed at baseline and in response to inflammatory challenge (LPS) using RNA-Seq. (**B**) Gene set enrichment analysis of HSPC^AML^ transcriptome at baseline shows highly enriched dysregulated inflammation-related pathways. (**C**) In response to an LPS challenge, HSPC^AML^ showed enhanced transcriptional activity. (**D**) Correlative RNA-Seq/ATAC-Seq analysis of AML-experienced, LPS-challenged HSPCs shows that 183 differentially gene expressed are also differentially accessible, with majority of these genes transcriptionally upregulated (**E**).

Our preliminary studies in C1498 grafts (Figure 2A) indeed suggest that residual healthy AML-experienced HSPCs (HSPC^AML^) develop a memory of sterile inflammation. Specifically, our transcriptomic data from C1498- experienced HSPC^AML^ showed differences in gene expression compared to naïve HSPCs that persisted 16 weeks after adoptive transfer to a leukemia-free niche. Gene set enrichment studies showed dysregulated inflammatory programs and metabolic pathway changes (Figure 2B), both defining traits of an immune memory [25]. AML experienced HSPCs also showed an aggravated response to an LPS-challenge, another characteristic of trained immunity (Figure 2C). Finally, preliminary correlative transcriptomic and chromatin accessibility (ATAC-Seq) analysis of LPS-challenged HSPC^AML^, identified 183 differentially expressed genes that also showed differential chromatin accessibility, suggesting a potential epigenetic imprint of AML exposure (Figure 2D, 2E). While further investigation is needed, our preliminary observations reveal aspects of innate immune reprogramming that raise further questions on whether AML-experienced HSPCs: 1) accelerate clonal selection and evolution of CH, 2) support the emergence of resistant MRD clones and relapse or 3) dysregulate the adaptive immune landscape. Clearly, a better understanding of a durable inflammatory legacy and the long-term functional consequences for AML patients in remission is needed.
